# Network Pharmacology-Based Approach Uncovers the Mechanism of GuanXinNing Tablet for Treating Thrombus by MAPKs Signal Pathway

**DOI:** 10.3389/fphar.2020.00652

**Published:** 2020-05-13

**Authors:** Mu-Lan Wang, Qin-Qin Yang, Xu-Hui Ying, Yuan-Yuan Li, Yang-Sheng Wu, Qi-Yang Shou, Quan-Xin Ma, Zi-Wei Zhu, Min-Li Chen

**Affiliations:** ^1^Academy of Chinese Medicine & Institute of Comparative Medicine, Zhejiang Chinese Medical University, Hangzhou, China; ^2^The Department of Medicine, Chiatai Qingchunbao Pharmaceutical Co., Ltd., Hangzhou, China; ^3^Department of Experimental Animals, Zhejiang Academy of Traditional Chinese Medicine, Hangzhou, China

**Keywords:** GuanXinNing tablet, network pharmacology, thrombus, Danshen, Chuanxiong, MAPKs signal pathway

## Abstract

**Background:**

GuanXinNing tablet (GXNT), a traditional Chinese patent medicine, has been found to have remarkable antithrombotic effects and can effectively inhibit pro-thrombotic factors in previous studies. However, the mechanism of its antithrombotic effects remains little known.

**Methods:**

In this study, we first determined and identified the sources of each main compound in GXNT using liquid chromatography-mass spectrometry (LC-MS). Through the approach of network pharmacology, we predicted the action targets of the active components, mapped the target genes related to thrombus, and obtained potential antithrombotic targets for active ingredients. We then performed gene ontology (GO) enrichment analyses and KEGG signaling pathway analyses for the action targets, and constructed networks of active component–target and active component–target–pathway for GXNT. Additionally, we evaluated the pharmacodynamic effects of GXNT on thrombus using the rat thrombus model induced by FeCl_3_, observed the effects of antiplatelet aggregation *via* platelet assay, and further verified the results predicted by network pharmacology *via* Western blot.

**Results:**

In total, 14 active ingredients were identified in GXNT, and 83 action targets were predicted, 17 of which are antithrombotic targets that potentially participate in processes including response to oxidative stress and positive regulation of blood vessel endothelial cell migration. KEGG pathway analyses revealed that the predicted action targets were involved in multiple signal pathways, such as MAPK, IL-17, and platelet activation. Pharmacodynamics study found that GXNT could significantly reduce the thrombus length and weight, lower platelet aggregation function, and decrease the levels of Fbg and PAI-1. In addition, GXNT could significantly increase 6-keto-PGF1α content and regulate the ratio of TXB_2_/6-keto-PGF1α, while not having dramatic effects on TXB_2_. GXNT was also observed to visibly inhibit maximum platelet aggregation. Herein, we further studied the thrombus-related MAPKs signaling pathway and found that GXNT could significantly reduce the phosphorylation levels of p38MAPK, ERK, and JNK proteins in platelet.

**Conclusions:**

This study revealed the pharmacodynamic material basis of GXNT and its potential multicomponent–multitarget–multipath pharmacological effects, confirmed the antithrombotic effects of GXNT, and showed that its mechanism may be related to inhibiting phosphorylation of p38, ERK, and JNK proteins in MAPKs signaling pathway, partially verifying the results from network pharmacology. The results from this study could provide a theoretical basis for the development and clinical application of GXNT.

## Introduction

Thrombus is a common pathophysiological basis for various cardiovascular diseases in the clinic, such as acute myocardial infarction, stroke, and coronary heart disease (Sadowski et al., 2014; Wang, 2018). Traditional Chinese medicine (TCM) believes that cold coagulation and blood stasis plays an important role in thrombotic diseases (Gu, 2010). Warming collaterals and activating blood circulation therapy is a general principle for treating cold coagulation and blood stasis syndrome, according to the Yellow Emperor's Internal Classic. As a consequence, Chinese medicine with the function of activating blood circulation and removing blood stasis is often used to prevent and cure thromboembolic diseases.

GuanXinNing is a classical Chinese herbal formula preparation, which is composed of two well-established Chinese herbs that activate blood circulation and remove blood stasis: *Salvia miltiorrhiza* Bge. (Chinese name Danshen, DS) and *Ligusticum chuanxiong* Hort. (Chinese name Chuanxiong, CX). This preparation has the effect of activating blood circulation, removing blood stasis, dredging arteries, and nourishing the heart. To improve patient convenience and compliance, GuanXinNing tablet (GXNT) is a novel preparation developed from the widely used GuanXinNing injection with an improved extraction process. GXNT consists of extracts from Danshen and Chuanxiong at the ratio of 1:1 (Chen et al., 2005), and has already been approved for listing by the China Food and Drug Administration (CFDA approval no. Z20150028). Danshen, the dry roots of *Salvia miltiorrhiza* Bge., is beneficial to heart and liver with a bitter taste and a slightly cold property. Studies have demonstrated that Danshen has significant anti-arrhythmia effects *via* reducing myocardial infarct size, protecting myocardial injury (Chang et al., 2016), and improving myocardial ischemia (Zhang et al., 2013). The second Chinese herb component, Chuanxiong, is the dry rhizome of *Ligusticum chuanxiong* Hort. Chuanxiong is known to protect the liver, gallbladder, and pericardium with a mild property and an acrid taste, and has the effects of activating blood circulation, moving qi, dispelling wind, and relieving pain (Chen et al., 2018b). Modern pharmacological studies have shown that Chuanxiong has antioxidation, anti-inflammation, neuroprotection, and anti-bacteria activities (Chen et al., 2018b; Shan et al., 2018). Moreover, our previous studies have found that GXNT could reduce platelet aggregation, scavenge free radicals, ameliorate blood coagulation in rats with qi stagnation and blood stasis, protect the vascular endothelium (Chen et al., 2005), and have antithrombotic activities with multiple-target effects (Wang et al., 2016). Nevertheless, TCM is a complex chemical composition system of multiple components, with multiple targets, multiple links, and multiple effects. Therefore, a holistic view of “multiple components-multiple targets-multiple pathways” is needed to study the material basis and action mechanism of GXNT on thrombus.

Network pharmacology uses high-throughput omics data analysis, virtual computing, and network database retrieval to construct an interaction network of “compound-gene-disease” and to provide a holistic understanding of the relationship between drugs and targets. Integrating with systems biology, multi-directional pharmacology and bioinformatics, and network pharmacology offers new approaches and strategies for designing and developing new drugs (Hopkins, 2008; Li, 2013). In particular, it has unique advantages and potential in predicting and identifying the active ingredient clusters and action targets of Chinese medicines, and in discovering new indications through active molecule screening, target prediction, network construction, and analysis. The systemic and holistic traits of network pharmacology are in line with the complexity of TCM, making it widely adopted in studying the pharmacodynamic material basis and action mechanism of TCM preparations, such as XinShengHua granule (Pang et al., 2018), MaZiRen wan (Huang et al., 2018), YinHuangQingFei capsule (Yu et al., 2017), YangXinShi tablet (Chen et al., 2018a), etc.

In this study, we used network pharmacology to predict the targets of active ingredients in GXNT and investigate its action mechanism. Firstly, the main active components of GXNT were identified and screened based on liquid chromatography-mass spectrometry (LC-MS) combined with traditional Chinese medicine system pharmacology technology platform (TCMSP). Then, active ingredient targets were predicted using Swiss Target Prediction web server to construct the active ingredient-target, protein interaction, and component–target–pathway network for analyzing the pharmacodynamic basis and action mechanism of GXNT. Next, the common carotid artery thrombus model in rats induced by FeCl_3_ was adopted to further verify the antithrombotic effects of GXNT, followed by the antiplatelet study. Finally, we examined the protein expressions in the predicted thrombus-related signaling pathways *via* Western blot to verify the antithrombotic mechanism of GXNT. The results from this study provided a theoretical reference for the development and utilization of GXNT.

## Materials and Methods

### Materials and Regents

GXNT (GXN extract powder, raw drug dosage of 12.8 g/g), Danshen and Chuanxiong were all provided by Chiatai Qinchunbao Pharmaceutical co., LTD. (Hangzhou, China). The herbal medicines of Danshen and Chuanxiong in GXNT were collected from Linyi City (Shandong Province, China) and Dujiangyan City (Sichuan Province, China) respectively, and were authenticated correspondingly by Prof. Yuqing Ye (Chinese Medicine Resource Research and Development Center, Shanghai Institute of Traditional Chinese Medicine) and Prof. Guihua Jiang (School of Pharmacy, Chengdu Chinese Medical University). The voucher specimens were deposited in the Quality Department of Chiatai Qinchunbao Pharmaceutical co., LTD. Reference standards of tanshinol sodium (110855-200809, purity=100%), protocatechualdehyde (110810-201608, purity=99.3%), chlorogenic acid (110753-200413, purity=100%), caffeic acid (110885-200102, purity=100%), ferulic acid (110773-201012, purity=100%), rosmarinic acid (111871-201505, purity=98.5%), and salvianolic acid B (111562-201514, purity=93.7%) were all purchased from National Institute for the Control of Pharmaceutical and Biological Products (Beijing, China). LC-MS grade acetonitrile and formic acid with ≥98.0% of the purity were purchased from Merck (Darmstadt, Germany). Other reagents were all of analytical grade. Ultrapure water purified by Millipore Ultra-pure Water Purifier (Millipore, Milford, MA, USA) was used. Clopidogrel bisulfate was purchased from Sanofi-Aventis Pharmaceutical Co., Ltd. (Hangzhou, China). Fibrinogen (Fbg) kit was purchased from Dade Behring Marburg GmbH (Marburg, Germany). Plasminogen activator inhibitors (PAI-1), 6-keto-prostaglandin F1α (6-keto-PGF1α), and thromboxane B_2_ (TXB_2_) assay kits were all purchased from Nanjing Jiancheng Bioengineering Research Institute Co., Ltd. (Nanjing, China). Aspirin enteric-coated tablets were purchased from Bayer healthcare Co., Ltd. (Leverkusen, Germany). Adenosine diphosphate (ADP) and albumin from bovine serum (BSA) were purchased from Sigma-Aldrich (St. Louis, Missouri, USA). KeyGEN total protein extraction kit, BCA protein assay kit and western stripping buffer were purchased from Beyotime Biosciences (Shanghai, China). Primary antibodies against Phospho-p44/42 MAPK (Erk1/2, #4370), Phospho-p38 MAPK (#4511), Phospho-SAPK/JNK (#9255), and GAPDH were all purchased from Cell Signaling Technology (Boston, MA, USA). All male Sprague-Dawley (Gfeller et al.) rats, weighing 300 to 350 g, were purchased from Shanghai SLAC Laboratory Animal Co., Ltd (Certification No: SCXK [Hu] 2012-002; Shanghai, China). Prior to the experiment, the animals were housed in individually ventilated cages (IVC) with two rats in each cage under a 12-h light/dark cycle, and were provided with food and water *ad libitum*. All experiments were carried out strictly according to the requirements of the Institutional Animal Care and Use Committee of Zhejiang Chinese Medical University, and was approved by the Laboratory Animal Research Center of Zhejiang Chinese Medical University (Certification No: SYXK [Zhe] 2013-184).

### Preparation for the Control Sample

The appropriate amount of each standard sample was accurately weighed using 1/100,000 precision analytical balance (Sartorius Group, German), and was diluted with 50% methanol solution to a constant volume for preparing mother liquor. It was then diluted to a series of concentrations and filtered with a 0.45-μm needle filter, from which the filtrate was obtained finally.

### Preparation for GXNT Testing Sample

We took a mixture of Danshen and Chuanxiong (10 kg each), added it with 160 L water, and boiled it for 2 h. Afterwards, the extracted solution was poured out, and the remaining residue was extracted twice, in which 120 L water was added each time and boiled for 1.5 h. The three extracts were merged to concentrate to 15 L at 60°C in a single-effect concentrator, and the solution after concentration was transferred to a rotary evaporator to concentrate to approximately 9 L at 60°C. About 35 L of 95% ethanol solution was added to the concentrated liquor, and was allowed to stand overnight. The supernatant was taken and concentrated to about 5 L at 60°C using a rotary evaporator. The concentrate was placed into a vacuum drying oven, dried thoroughly at 60°C, and powdered homogeneously with a powder machine to obtain solid powder of about 1.5 kg (raw dose of 12.85 g/g). Subsequently, 1.0 g of the powder was accurately weighed, brought to 20 mL with 50% methanol added, and sonicated for 10 min. It was then filtered through a 0.45-μm microporous filter column, from which an appropriate amount was injected into HPLC-MS instrument for analysis (Shimadzu LC-20A liquid chromatograph, Shimadzu, Japan; API 3200 LCMS/MS Mass Spectrometry System, American AB SCIEX, USA).

### Preparation for Danshen Testing Sample

The preparation was performed in accordance with GXNT technology. We took 6 kg of Danshen and decocted it with water three times. In specific, 48 L was added and boiled for 2 h for the first time, and 36 L was added and boiled for 1.5 h for the second and third time. The three extracts were merged to concentrate to 3.2 L at 60°C in a single-effect concentrator. About 8.5 L of 95% ethanol solution was added into the concentrated liquor, and was allowed to stand overnight. The supernatant was taken and concentrated to about 1 L at 60°C using a rotary evaporator. The concentrate was placed into a vacuum drying oven, dried thoroughly at 60°C, and powdered evenly to obtain about 402 g of solid powder (raw dose of 15.0 g/g). Subsequently, 0.5 g of the powder was weighed accurately, brought to 20 mL with 50% methanol, and sonicated for 10 min. After that, it was filtered through a 0.45-μm microporous filter column, from which an appropriate amount was injected into HPLC-MS instrument for analysis.

### Preparation for Chuanxiong Testing Sample

The preparation was conducted according to GXNT technology. We took 3.5 kg of Chuanxiong, and decocted it with water three times. In specific, 28 L was added and boiled for 2 h for the first time, and 21 L was added and boiled for 1.5 h for the second and third time. The three extracts were merged to concentrate to 2 L at 60°C in a single-effect concentrator. About 7.5 L of 95% ethanol solution was added into the concentrate, and was allowed to stand overnight. The supernatant was taken and concentrated to about 1 L at 60°C using a rotary evaporator. The concentrate was placed into a vacuum drying oven, dried thoroughly at 60°C, and powdered evenly to obtain about 538 g of solid powder (raw dose of 6.5 g/g). Subsequently, 1.0 g of powder was weighed accurately, brought to 20 mL with 50% methanol, and sonicated for 10 min. After that, it was filtered through a 0.45-μm microporous filter column, from which an appropriate amount was taken to inject into HPLC-MS instrument for analysis.

### LC-MS Analysis

LC-MS analysis of samples was carried out on a Shimadzu LC-20A liquid chromatograph (Shimadzu, Japan). An Agilent ZORBAX SB-C18 column (250×4.6 mm i.d., 5 μm, Agilent, USA) was used for column separation. The column temperature was maintained at 40°C, and the flow rate was kept at 1 mL/min, with acetonitrile as the mobile phase A and 0.1% formic acid in water as the mobile phase B. The gradient running procedure was programmed as follows: 0~5 min, 40~40% A; 5~25 min, 40~69% A; 25~30 min, 69~100% A. The injection volume was 5 μl. In addition, the mass spectrometer was an API 3200 LCMS/MS system. The detection mode was Q1 scan profile mode. The total scan time was 5 s per cycle with 599 cycles, and data was collected in the positive mode. The capillary voltage was 4500 V, and the mass range was from m/z 100 to 1000. Curtain Gas, Atomized Gas (Gas1), and Auxiliary Gas (Gas2) were nitrogen, and the pressure was set to 15 psi, 30 and 30 psi, respectively. We used 4500 V for the spray voltage, 450°C for the atomization temperature, 10 V for the collision chamber inlet voltage, and 70 V for the de-clustered voltage (DP). All data collection and processing were performed using Analyst software (version 1.6). The chemical structures of main compounds identified in GXNT were drawn with ChemDraw from CambridgeSoft. The chemical drawing software is capable of performing accurate mass analyses for LC/MS (electrospray), such as adducts and protonated molecules.

### Establishment of SMILES Format File for Active Ingredients in GXNT

The molecular structure of the active compound was mapped with ChemBio Draw Ultra 14.0 software, and was saved in the MDL sdf. format. All chemical structures were converted to Mol2 format using ChemBio 3D Ultra software in order to establish an active molecular library. The active molecular Mol2 file was converted to a SMILES file using Open Babel GUI software for subsequent analyses.

### Prediction of Potential Targets for Active Ingredients in GXNT

Swiss Target Prediction (http://www.swisstargetprediction.ch/) is a web server to accurately predict the action targets of bioactive molecules based on the similarity of two dimension and three dimension of known ligands, providing valuable insights into the action mechanism of active molecules (Gfeller et al., 2014). The SMILE format files of active ingredients identified by LC-MS were uploaded to the Swiss Target Prediction server, and “Homo sapiens” was selected as the species. Then, the potential drug targets were searched using the active small molecules as probes. The target prediction results were sorted from high to low according to “Probability”, and the official names of drug targets were retrieved through the UniProtKB search function in the UniProt database (http://www.uniprot.org/).

### Mapping of Thrombus-Related Targets

Reported genes, possibly related to thrombus, were searched by the keyword “thrombus” in CooLGeN (http://ci.smu.edu.cn/CooLGeN/) and in the GeneCards database. Comparing these with the targets obtained from Swiss Target Prediction server, we obtained the potential targets of the active ingredients in GXNT that are potentially involved in the antithrombotic mechanism.

### Functional Enrichment Analysis of the Potential Action Targets

Bioscape Annotation Database Metascape (http://metascape.org) is a reliable, effective, and intuitive online bioinformatics annotation tool for understanding the biological functions of genes and protein lists on a large scale for biomedical researchers. Gene ontology (GO) enrichment and KEGG pathway annotation analyses of the basic ontology term were performed using Metascape for the potential targets of GuanXinNing, and “*P* < 0.05” was considered as the statistically significant screening condition.

### Construction of Component–Target Network and Component–Target–Pathway Network

The action targets and related signaling pathways were predicted according to the active component candidates identified from GXNT, and were then imported into Cytoscape software for constructing compound-target networks, and compound-target-path networks to explore the overall pharmacological mechanisms of GXNT. The importance of every node in the network was determined by the degree of topological parameters. The degree of a node refers to the number of edges connected to that node, i.e. the higher the degree is, the more nodes it is directly connected to, and the more importance the node has in the network. Edges represent the interactions between the compounds and the targets in the network.

### Thrombus Animal Experiment

#### Animal Administration and Modeling

After 3 to 5 days of adaptive breeding, 48 SD rats were randomly divided into six groups, namely, the control group, the model group, the GXNT low, medium, and high groups with doses of 75, 150, and 300 mg/kg, and the positive group (n = 8). Each GXNT group was given the corresponding dose of GXN extract powder solution by oral administration. The positive group was given 12.5 mg/kg of clopidogrel solution orally, and the control group and the model group were intragastrically administrated with 10 mL/kg of distilled water. After 1 h of administration, thrombus model operation induced by FeCl_3_ was conducted in SD rats. Briefly, rats were anesthetized by intraperitoneal injection of 3% sodium pentobarbital solution (0.15 mL/kg). The rats were fixed on a 37°C insulated operating platform with neck hair shaved and neck skin disinfected. Next, the right common carotid artery was carefully separated, and a plastic paper with a width of 1 cm was placed on the bottom of the right common carotid artery. Then, the 1 ×1 cm filter paper, added with 10 µl of 35% FeCl_3_, was wrapped around the common carotid artery rapidly for 15 min of external application. Afterwards, we removed the plastic paper and the filter paper, ligated both ends of the thrombus, and cut the embolus.

#### Measurement of Thrombus Length and Weight

Before SD rats were sacrificed, the emboli of the rats were quickly cut, and the redundant blood was absorbed by clean filter paper. The length of the thrombus was accurately measured using vernier caliper and recorded as L_right_. The weight of the thrombus was also accurately weighed with an analytical balance and recorded as M_right_. Then, a proper length of the left common carotid artery of SD rats was taken, with the blood in the vessels absorbed by filter paper. The length of the blood vessel was precisely measured by the vernier caliper and recorded as L_left_. The weight of the thrombus was weighed using an analytical balance and recorded as M_left_. The weight of the thrombus was calculated with the following formula:

$$\frac{L\left(left\right)}{M\left(left\right)}=\frac{L\left(right\right)}{M\left(right\right)-M\left(thrombus\right)},M\left(thrombus\right)=M\left(right\right)-\left(\frac{M\left(left\right)}{L\left(left\right)}\right)\times L\left(right\right)$$

#### Preparation of Platelet-Rich Plasma and Platelet-Poor Plasma

Blood of rats in each group was taken from the abdominal aorta, transfused into PE tubes containing 3.8% sodium citrate anticoagulant (9:1, v/v), and then repeatedly inverted several times to fully mix the blood and anticoagulant. Part of the anticoagulant blood in the PE tube was centrifuged at 3,500 rpm for 15 min, and plasma was taken and stored at −80°C for further usage. The rest of the anticoagulant blood was taken and centrifuged at 1000 rpm for 10 min at room temperature, and the supernatant, namely platelet-rich plasma (PRP), was obtained to detect platelet aggregation rates and protein expressions. The remaining part was further centrifuged at 3000 rpm for 10 min, and the resulting supernatant, namely platelet-poor plasma (PPP), was taken.

#### Determination of Fbg, PAI-1, 6-keto-PGF1α, and TXB_2_ in the Blood

Anti-coagulated plasma at −80°C was taken and thawed. Fbg was measured using CA500 automatic blood coagulation analyzer (Sysmex, Japan). The specific procedures were carried out in strict accordance with the commercially available kit. The PAI-1, 6- 6-keto-PGF1α, and TXB_2_ assay kits were used to detect the expression levels of PAI-1, 6-keto-PGF1α, and TXB_2_ in samples by the enzyme linked immunosorbent assay (ELISA) method under the guidance of corresponding kit instructions.

#### Platelet Aggregation Assay

According to optical principles, the platelet aggregation rate was measured using Chrono-log platelet aggregation instrument (CHRONO-LOG, USA). We took PRP and PPP into turbidity tubes, and placed them in preheating holes. Platelet aggregation assay was performed after incubation at 37°C for 5 min, and PPP was applied to zero setting during measurement. Then, ADP (10 μM) as the agonist was added to PRP with magnetic stirrer stirring, and the aggregation curve was traced. The inhibition rate of platelet aggregation was calculated by the following formula:

Inhibition rate (%)={(aggregation rate of the control group−aggregation rate of the administration group)/aggregation rate of  the control group}×100%

#### Determination of MAPKs Signaling Pathway-Related Protein Expressions in Platelets by Western Blot

Prepared PRP was taken, induced with ADP, incubated for 20 min at 37°C, and centrifuged at 3000 rpm for 5 min. The supernatant was discarded, and the precipitated fraction was used for the extraction of total platelet protein, which was carried out according to the instructions of the KeyGEN total protein extraction kit. Then, protein concentrations were quantified using the BCA protein assay kit. After protein samples were mixed with sample loading buffer (4:1, v/v), they were boiled for 5 min. Next, proteins (10 μg) were separated by SDS-PAGE electrophoresis, and transferred to PVDF membranes. The membranes were blocked with 3% albumin from bovine serum at room temperature and then incubated overnight with primary antibodies (p-P38, p-ERK1/2, and p-JNK of 1:1000 dilution; GAPDH of 1:200 dilution; all diluted with 3% BSA) at 4°C. After 48 h, the membranes were washed with TBST for 4 times, and were incubated with the secondary antibodies at 37°C for 2 h. Finally, the membranes were washed with TBST for four times and with TBS for 1 min, and were scanned in the odyssey infrared fluorescence scanner (Thermo company, USA). Afterwards, the membranes containing p-P38, p-ERK1/2, p-JNK proteins were washed with western stripping buffer to further detect P38, ERK1/2, and JNK proteins by the procedures as mentioned above.

### Statistical Analysis

All data were statistically analyzed using SPSS 22.0 software, and expressed as mean ± standard error ($\bar{\chi }$± SEM). Statistical analysis was conducted *via* one-way analysis of variance (ANOVA) for comparison between groups and *via* L-S-D test for pairwise comparison. Drawings of statistical graph were done using GraphPad Prism 6.0 software. *P* < 0.05 indicates statistical significance.

## Results

### Optimization of Fingerprint of GXNT

**Figure 1:**
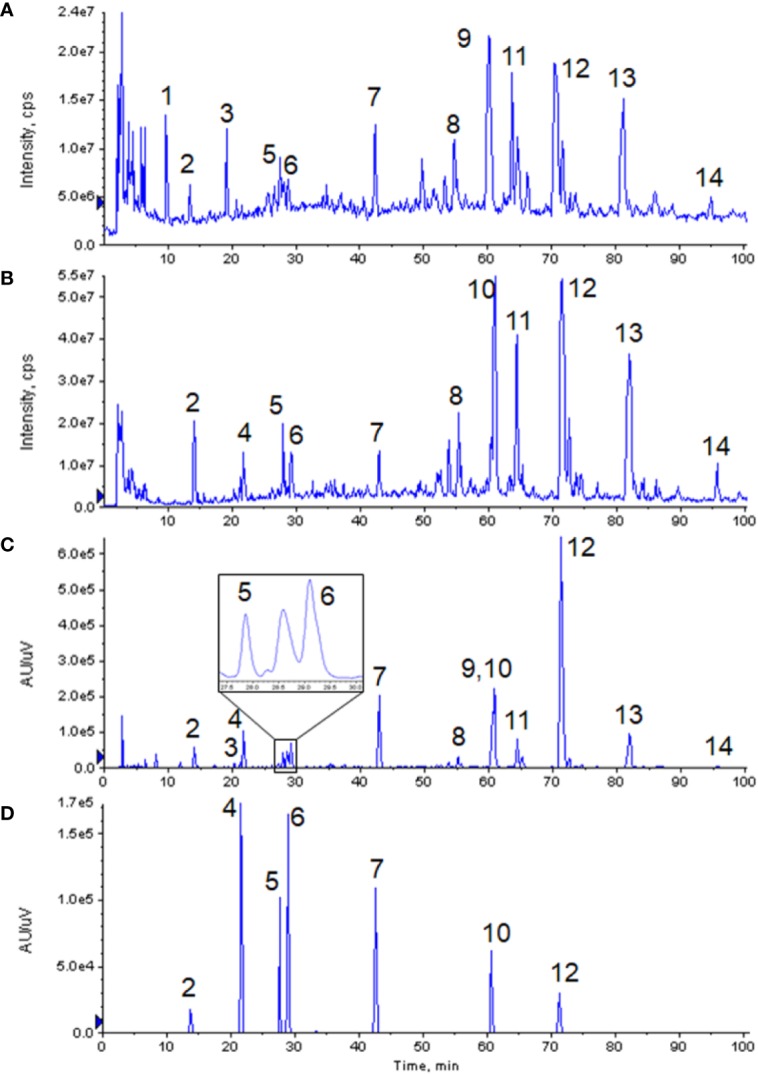
Chromatograms of GXNT and mixed standard. **(A)** TIC diagram of GXNT mass spectrometry in the positive mode. **(B)** TIC diagram of GXNT mass spectrometry in the negative mode. **(C)** Chromatogram of GXNT at 280 nm. **(D)** Chromatogram of mixed standard at 280 nm. (1) Phenylalanine; (2) Tanshinol; (3) Senkyunolide B; (4) Protocatechualdehyde; (5) Chlorogenic acid; (6) Caffeic acid; (7) Ferulic acid; (8) Salvianolic acid D; (9) Senkyunolide I; (10) Rosemary acid; (11) Isosalvianolic acid A; (12) Salvianolic acid B; (13) Salvianolic acid A; (14) Isosalvianolic acid C.

Non-volatile phosphoric acid was used as the mobile phase additive in the fingerprint of GXNT, which was established by the institute previously (Lin et al., 2017). Since this condition is not suitable for LC-MS system, the analytical method needs to be properly optimized. When no additive was used (i.e. pure water was used as the water phase), the compound had a wider peak shape, poor symmetry, and a certain degree of tailing. After a certain amount of formic acid was added, the peak shape could be visibly improved, and the mass spectral response was also enhanced. Hence, 0.1% formic acid was used as the aqueous phase. The optimized analysis conditions were used to analyze the GXNT samples. The mass spectrum TIC map (in positive and negative modes) and the ultraviolet chromatogram at 280 nm are both shown in **Figure 1**. Since the mass spectrometry detector was a broad-spectrum detector, many compounds that were limited to ultraviolet response could be detected, and thus more complete material information could be obtained. It was observed that the main ion peaks of TIC images under the positive and negative modes of the mass spectrum were all reflected in the fingerprint, indicating that the currently established fingerprint map basically satisfied the principle of compound information maximization.

### Maps of GXNT and Single Chinese Herb

**Figure 2:**
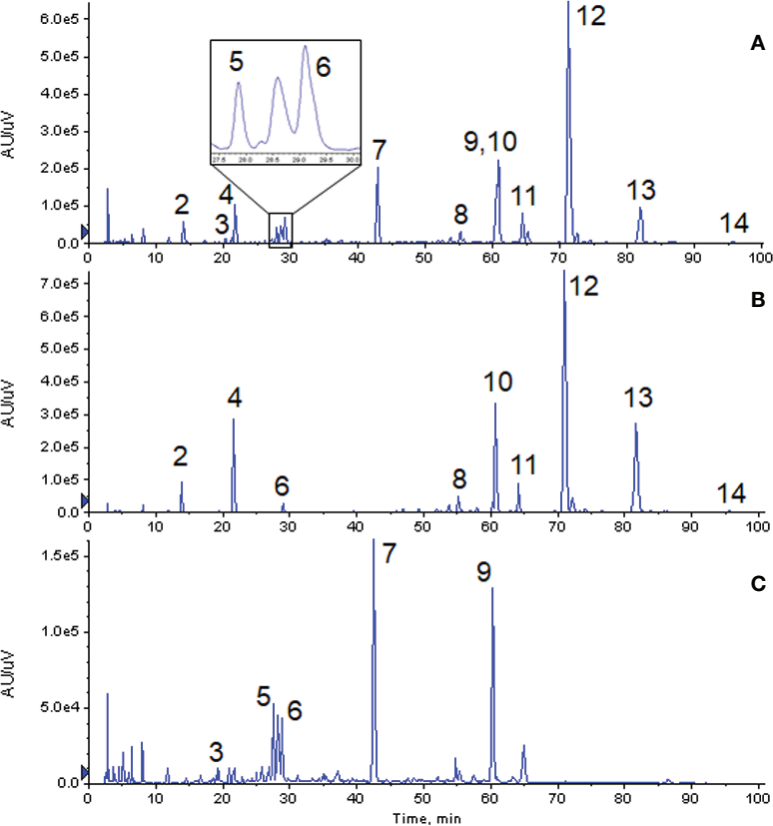
Comparison of chromatogram of each extract at 280 nm. **(A)** Ultraviolet chromatogram of GXNT. **(B)** Ultraviolet chromatogram of Danshen. **(C)** Ultraviolet chromatogram of Chuanxiong. (2) Tanshinol; (3) Senkyunolide B; (4) Protocatechualdehyde; (5) Chlorogenic acid; (6) Caffeic acid; (7) Ferulic acid; (8) Salvianolic acid D; (9) Senkyunolide I; (10) Rosemary acid; (11) Isosalvianolic acid A; (12) Salvianolic acid B; (13) Salvianolic acid A; (14) Isosalvianolic acid C.

From the chromatographic comparative map at 280 nm (as shown in **Figure 2**), it could be seen that most compounds in GXNT were from Danshen, while a small amount came from Chuanxiong, since most components in Chuanxiong were volatile oils and the extraction rate of the water extraction process was lower. Among all the compounds, peaks 1 and 6 were the common peaks of the two drugs. Though the fingerprint had only one peak at 60 min, we can see from the mass spectrometry of Danshen (peak 10) and Chuanxiong (peak 9) separately that both compounds had peaks at the same retention time, meaning that the single peak consists of two superimposed peaks from Danshen and Chuanxiong and that these two compounds are not the same substance. This is difficult to see in the ultraviolet chromatogram alone. However, it could be effectively identified in the mass spectrum, and distinguished and quantified separately by extracting ion peaks.

### Identification of Active Ingredients in GXNT

**Figure 3:**
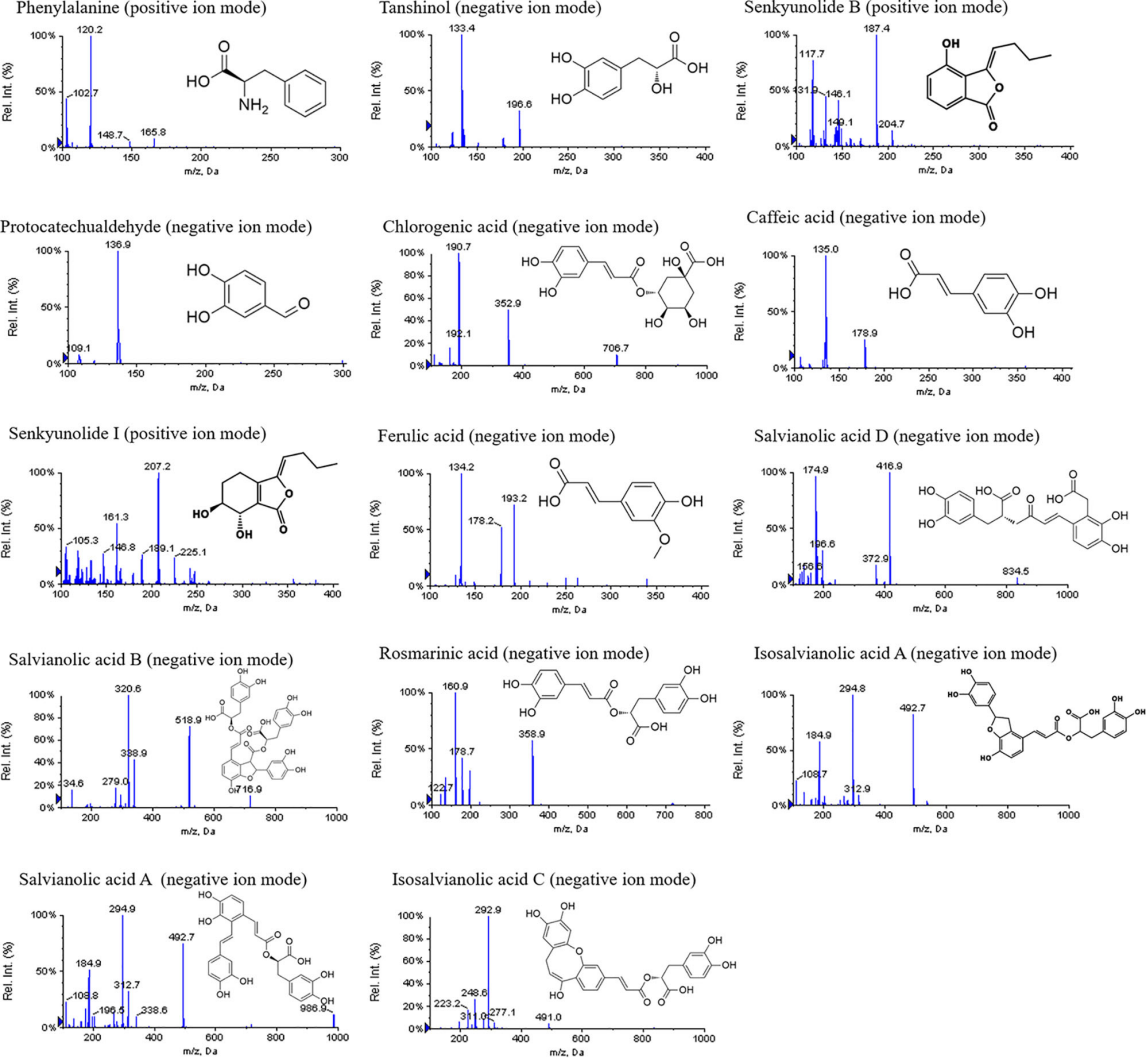
Mass spectrums and chemical structures of main compounds in GXNT.

The mass spectrometry information of the materials obtained from the experiment was compared with the related literature reports. As a result, 14 compounds were identified in GXNT, of which 7 major compounds were verified by standard products. The specific identification results were summarized in **Table 1**, and the mass spectrum and the structure of each compound are shown in **Figure 3**. In specific, peak 1 was identified as phenylalanine, in which one molecule of NH_3_ and/or HCOOH was removed to form a responsive fragment ion in the mass spectrum (Ying et al., 2013b). Peak 2 was identified as tanshinol, which responded weakly in the positive mode, with one molecule of H_2_O as well as HCOOH removed to form the major fragment ion in the negative mode (Chen et al., 2011). Peak 3 only responded in the positive mode of mass spectrometry, mainly forming a dehydrated ion peak. Senkyunolide B and C, corresponding to molecular weights and compounds that could form dehydrated ion peaks in Chuanxiong, were mainly obtained *via* searching the literature. Given the polarity according to the peak time and the structure of the compound, it was preliminarily presumed to be senkyunolide B (Hu et al., 2012). Peak 4 was identified as protocatechuic aldehyde, which responded only in the negative mode, where one molecule of CO was removed to form the major fragment ion of *m/z* 109 (Chen et al., 2011). Peak 5 was identified as chlorogenic acid, which mainly formed [M+H-192]+ by removing one molecule of quinic acid in the positive mode and fragment ions of quinic acid (*m/z* 191) in the negative mode (Ying et al., 2013a). Peak 6 was identified as caffeic acid, where molecule of H_2_O was removed in the positive mode of mass spectrometry and one molecule of CO_2_ was removed in the negative mode, corresponding to the carboxyl group of structure (Chen et al., 2011). Peak 7 was identified as ferulic acid, which could remove one molecule of H_2_O, CO, and CH_3_OH to form corresponding fragment ions in the positive mode, remove CH_3_• in the methoxy group on the benzene ring to form radical ions of *m/z* 178 in the negative mode, and remove CO_2_ of carboxyl group on the side chain to form the fragment ion of *m/z* 134 (Hu et al., 2012). Peak 8 was identified as salvianolic acid D, of which the main fragment ions were formed by firstly removing one molecule of CO_2_ and then removing one molecule of tanshinol (198 of molecular weight) in the positive and negative modes of mass spectrometry (Chen et al., 2011). Peak 9 was identified as senkyunolide I, of which the dehydrated ion peak (*m/z* 207) was obtained by removing one hydroxyl group from cyclohexane in the positive mode and the corresponding fragment ions were obtained by further removing the second hydroxyl group on the ring or the ester bond in the lactone ring (Hu et al., 2012). Peak 10 was identified as rosmarinic acid, and its structure was formed by the condensation of one molecule of caffeic acid and one molecule of tanshinol. Therefore, it could remove one molecule of caffeoyl group (162 of molecular weight) or caffeic acid (180 of molecular weight), and a molecule of tanshinol (198 of molecular weight) to form each fragment ion in the mass spectrometry (Chen et al., 2011). Peaks 11, 12, 13, and 14 were identified as isosalvianolic acid A, salvianolic acid B, salvianolic acid A, and isosalvianolic acid C, respectively. These compounds were similarly cleaved in the mass spectrometry, with single or multiple molecules of tanshinol (198 of molecular weight) removed to form each of the major fragment ions (Chen et al., 2011).

**Table 1:** Identification results of main compounds in GXNT.

Peak No.^a^	RT (min)	Main ions in the positive mode	Main ions in the negative mode	Molecular weight	Identification result	Source
1	9.7	166[M+H]+;149[M+H-NH3]+;120[M+H-HCOOH]+;103[M+H-HCOOH-NH3]+	164[M-H]−;147[M-H-NH3]−	165	Phenylalanine	Common
2	14.1	199[M+H]+	197[M-H]-;179[M-H-H2O]-;133[M-H-H2O-HCOOH]-	198	Tanshinol^c^	Danshen
3	19.2	205[M+H]+;187[M+H-H2O]+	n.d.^b^	204	Senkyunolide B	Chuanxiong
4	21.7	n.d.	137[M-H]-;109[M-H-CO]-	138	Protocatechualdehyde^c^	Danshen
5	27.9	355[M+H]+、163[M+H-192]+	707[2M-H]-;353[M-H]-;191[M-H-162]-	354	Chlorogenic acid^c^	Chuanxiong
6	29.1	181[M+H]+;163[M+H-H2O]+	179[M-H]-;135[M-H-CO2]-	180	Caffeic acid^c^	Common
7	42.9	195[M+H]+;177[M+H-H2O]+;149[M+H-H2O-CO]+;117[M+H-H2O-CO-CH3OH]+	193[M-H]-;178[M-H-CH3·]-•;134[M-H-CH3·-CO2]-•	194	Ferulic acid^c^	Chuanxiong
8	55.3	419[M+H]+;177[M+H-CO2-198]+	835[2M-H]-;417[M-H]-;373[M-H-CO2]-;175[M-H-CO2-198]-	418	Salvianolic acid D	Danshen
9	60.6	225[M+H]+;207[M+H-H2O]+;189[M+H-H2O-H2O]+;161[M+H-H2O-HCOOH]+	n.d.	224	Senkyunolide I	Chuanxiong
10	60.9	361[M+H]+;181[M+H-180]+;163[M+H-198]+	359[M-H]-;197[M-H-162]-;179[M-H-180]-;161[M-H-198]-	360	Rosemary acid^c^	Danshen
11	64.4	495[M+H]+;297[M+H-198]+	493[M-H]-;295[M-H-198]-	494	Isosalvianolic acid A	Danshen
12	71.3	719[M+H]+;521[M+H-198]+;323[M+H-198-198]+	717[M-H]-;519[M-H-198]-;321[M-H-198-198]-	718	Salvianolic acid B^c^	Danshen
13	81.9	495[M+H]+;297[M+H-198]+	987[2M-H]-;493[M-H]-;295[M-H-198]-	494	Salvianolic acid A	Danshen
14	95.6	n.d.	491[M-H]-;293[M-H-198]-	492	Isosalvianolic acid C	Danshen

^a^Peak No. is consistent with the label of **Figure 1**; ^b^n.d. (not detected) means not detected, no or below the detection limit; ^c^Compound has been confirmed by reference standard.

### Content Quantification of Seven Main Active Ingredients in GXNT

We took two samples each of GXNT, Danshen and Chuanxiong herbs in parallel, calculated the amounts of the seven compounds confirmed by reference standards using the corresponding standard curves listed in **Table 2**, and investigated the changes of the seven compounds in each single Chinese herb and the Chinese compound formula. The results are shown in **Table 3** in detail. In each extract, the seven compounds accounted for about 6.5% of the total amount of extract from GXNT. The amount of salvianolic acid B was much higher in the extract of GXNT and Danshen than that in the extract of Chuanxiong. The current quality standard of GXNT uses salvianolic acid B and ferulic acid to control the quality of Danshen and Chuanxiong, respectively. However, our results indicated that ferulic acid only accounts for 0.316% of the Chuanxiong extract, which is not enough to reflect the quality of the whole Chuanxiong extract. Therefore, the quality control indexes for Chuanxiong need to be improved. In terms of raw drug content, the amount of each compound is similar in the medicinal materials compared to that in the compound formula, with some individual compounds having a slightly lower amount in the compound formula.

**Table 2:** Standard curves of seven main compounds.

Peak No.	Compound name	RT (min)	Linear relation	R^2^	Linear range (μg/mL)
2	Tanshinol sodium	13.7	y = 0.031x - 0.0658	0.9999	40.8~916.0
4	Protocatechualdehyde	21.6	y = 0.2065x - 0.5731	0.9999	23.1~462.4
5	Chlorogenic acid	27.6	y = 0.1021x - 0.2181	0.9999	21.8~436.8
6	Caffeic acid	28.9	y = 0.2006x - 0.4056	0.9999	21.0~419.2
7	Ferulic acid	42.6	y = 0.1855x - 0.4126	0.9999	21.4~428.8
10	Rosmarinic acid^a^	60.7	y = 0.6489x + 5.0937	0.9744	40.2~803.2
12	Salvianolic acid B	71.3	y = 0.0673x - 0.1907	0.9999	198.4~3968

^a^Rosmarinic acid was quantified by extracting ion peaks by mass spectrometry.

**Table 3:** Determination results of seven main compounds.

Peak No.	Compound name	Content in extract (%)			Content of raw drug (%)		
		GXNT	Danshen	Chuanxiong	GXNT	Danshen	Chuanxiong
2	Tanshinol	0.580	1.882	n.d.	0.090	0.125	n.d.
4	Protocatechualdehyde	0.135	0.763	n.d.	0.021	0.051	n.d.
5	Chlorogenic acid	0.084	n.d.	0.111	0.013	n.d.	0.017
6	Caffeic acid	0.097	0.082	0.069	0.015	0.005	0.011
7	Ferulic acid	0.362	n.d.	0.316	0.056	n.d.	0.049
10	Rosmarinic acid	0.551	1.547	n.d.	0.086	0.103	n.d.
12	Salvianolic acid B	4.676	12.337	n.d.	0.728	0.822	n.d.
	Total	6.485	16.611	0.496	1.009	1.107	0.076

n.d. (not detected) means not detected, no or below the detection limit.

### Screening of Potential Antithrombotic Targets for Active Ingredients in GXNT

**Figure 4:**
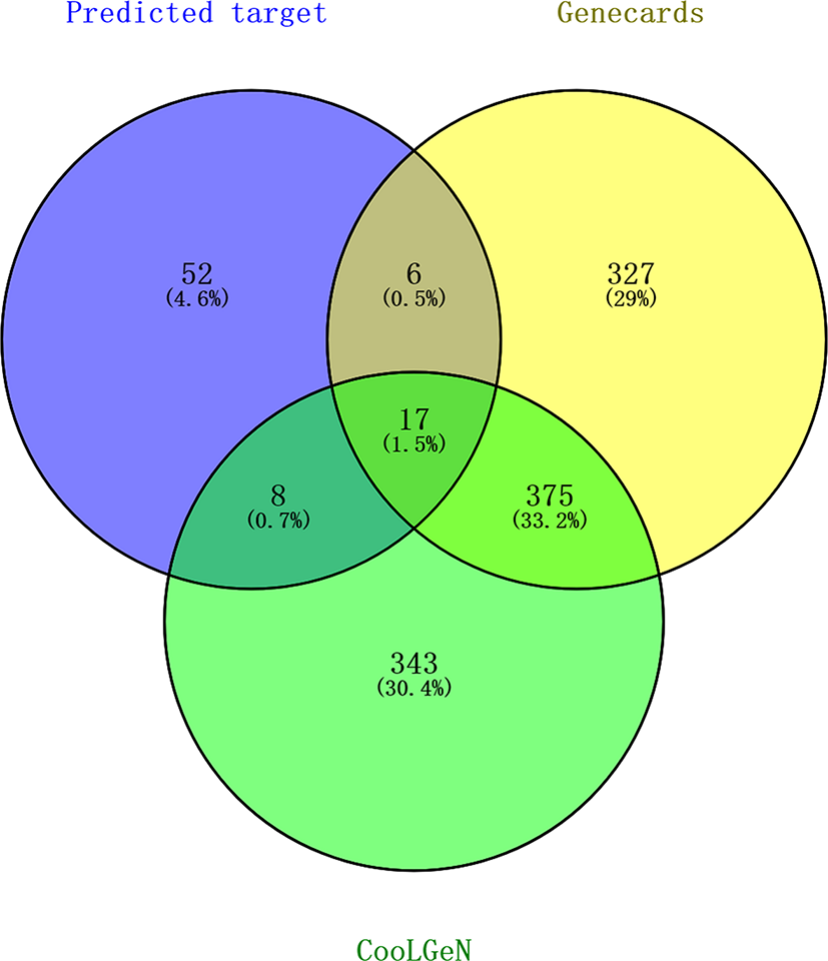
Screening of targets for the identified components of GXNT on thrombus. 83 Predicted targets (in the blue circle) were mapped to 743 thrombus-related targets in CooLGeN (in the green circle), and to 725 thrombus-related targets in the GeneCards database (in the yellow circle), respectively. Among them, 23 targets were found in the GeneCards database, and 25 targets were found in the CooLGeN database. The intersection of these targets resulted in a total of 17 potential antithrombotic targets of GXNT.

**Table 4** shows that different active compounds in GXNT could act on the same target, and the same active compound could also act on different targets, reflecting multi-component and multi-target action mode of GXNT. In specific, 83 unique targets were predicted from the 14 identified components using the Swiss Target Prediction analysis platform. The targets were also mapped to 743 targets possibly related to the occurrence and development of thrombus in the CooLGeN database, and to 725 targets associated with the occurrence and development of thrombus in the GeneCards database. Among them, 23 targets were also found in the GeneCards database, and 25 targets were found in the CooLGeN database. The intersection of these targets resulted in a total of 17 potential antithrombotic targets of GXNT (shown in **Figure 4**), including MAPT, EGFR, KDR, MMP2, MMP9, MMP13, MMP1, MMP10, PTPN1, FYN, SRC, PRKCA, PTGS1, PTGS2, JUN, EDNRA, and ALOX12.

**Table 4:** Potential targets from active ingredient candidates of GXNT.

Active ingredient candidates	Predicted targets
Phenylalanine	CA12, CA1, CA2, ALPL, CA3, ALPI, CA6, CA5A, CA7, CACNA2D1, CA9, CA14, PLAA, CA5B, CA13
Tanshinol	CA12, CA1, CA2, CA3, CA6, CA5A, CA7, CA13, TDP1, CA14, CA5B, MAPT, EGFR, ERBB2, LCK
Senkyunolide B	MBNL1, MBNL2, MBNL3, MAPT, CYP19A1, FLT1, FLT4, KDR, ESR1,ESR2, CDK1, CDK2, CDK4, CDK3, CDK6
Protocatechualdehyde	COMT, CA1, CA2, CA3, CA5A, CA7, CA5B, CA13, TYR, TDP1, MAPT, CA9, KDM4E, KDM4A, KDM4B
Chlorogenic acid	AKR1B10, AKR1B1, AKR1B15, AKR1A1, AKR1E2, MMP2, MMP9, MMP12, MMP13, MMP1, MMP3, MMP10, MMP27,MMP20, TDP1
Caffeic acid	CA12, CA1, CA2, CA3, PTPN2, PTPN1, CA6, CA5A, CA7, CA9, CA13,TDP1, CA14, CA5B, CA4
Ferulic acid	CA12, CA1, CA2, CA3, CA6, CA5A, CA7, CA9, CA13, CA14, CA5B, TDP1, AKR1B10, AKR1B1, AKR1B15
Salvianolic acid D	EGFR, ERBB2, ERBB4, ERBB3, FYN, YES1, FGR, SRC, FRK, ESR1, ESR2, MAPT, AKR1B10, AKR1B1, AKR1B15
Senkyunolide I	PRKCG, PRKCB, PRKCA, PRKCQ, PRKCD, PTGS1, PTGS2, RELA, REL, JUN, JUNB JUND, CRYZ, ADORA1, EDNRA
Rosmarinic acid	AKR1B10, AKR1B1, AKR1B15, TDP1, AKR1A1, AKR1E2, MMP1, MMP2, MMP3, MMP9 MMP12, MMP13, MMP10, MMP27, FYN
Isosalvianolic acid A	MMP1, MMP2, MMP3, MMP9,MMP8, MMP12, MMP13, MMP10, MMP27, AKR1B10, AKR1B1, AKR1B15, AKR1A1, AKR1E2, TDP1
Salvianolic acid B	MMP1, MMP2, MMP3, MMP9, MMP8, MMP12, MMP13, MMP10, MMP27, PTGS1 PTGS2, AKR1B10, AKR1B1, AKR1B15, AKR1A1
Salvianolic acid A	FYN, SRC, YES1, FGR, FRK AKR1B10, AKR1B1, AKR1B15, AKR1A1, AKR1E2, TDP1 MMP1, MMP2, MMP3, MMP9
Isosalvianolic acid C	AKR1B10, AKR1B1, AKR1B15, AKR1A1, AKR1E2, ALOX15, ALOX12, TOP1, TOP1MT EGFR, ERBB2, ERBB4, ERBB3, PTGS1, PTGS2

### Functional Pathway Notes of Active Component Candidate Targets in GXNT

We performed GO enrichment analysis for the above mentioned 17 candidate targets of the active ingredients from GXNT *via* Metascape biomolecular function annotation system, which includes the analysis of biological process, molecular function and cellular components, and KEGG pathway annotation. And the top 10 terms with *P* < 0.05 are shown in **Table 5**. According to the screening results, the candidate targets for the active components in GXNT were involved in the biological process of response to oxidative stress, response to toxic substance, and response to inorganic substance. In terms of the molecular function, the targets were mainly related to metalloendopeptidase activity, endopeptidase activity, and protein domain specific binding. As for the cellular components, the extracellular matrix and membrane microdomain were the main components related to the targets. In addition, 34 pathways were revealed from the KEGG pathway enrichment analysis, including TNF signaling pathway, IL-17 signaling pathway, focal adhesion, MAPK signaling pathway, and platelet activation. These findings suggest that GXNT may play an antithrombotic role by regulating multi-dimensional signal cascades. In specific, MAPK signaling pathway is known to play an important role in the signal transduction *in vivo* and in maintaining the body's biological metabolic balance (Lien et al., 2017; Manne et al., 2018). Therefore, MAPK signaling pathway was selected in this study to further explore the molecular mechanism of GXNT.

**Table 5:** Gene Ontology (GO) and pathway enrichment analysis for active component candidate targets of GXNT.

Category	Term	Number of the targets	P-value
GO Biological process	Response to oxidative stress	9	5.324E-12
	Positive regulation of epithelial cell migration	7	1.274E-11
	Response to inorganic substance	8	1.630E-09
	Circulatory system process	7	4.640E-08
	Regulation of mitochondrial membrane potential	4	1.496E-07
	Positive regulation of blood vessel endothelial cell migration	4	2.702E-07
	Response to toxic substance	6	1.001E-06
	Superoxide anion generation	3	1.559E-06
	Cellular response to amino acid stimulus	3	1.289E-05
GO Molecular Function	Metalloendopeptidase activity	5	7.598E-09
	Ephrin receptor binding	3	8.383E-07
	Metallopeptidase activity	5	1.322E-07
	Endopeptidase activity	5	9.480E-06
	Peptidase activity, acting on L-amino acid peptides	5	4.938E-05
	Peptidase activity	5	5.929E-05
	Serine-type endopeptidase activity	3	0.0001949
	Serine-type peptidase activity	3	0.0002867
	Serine hydrolase activity	3	0.0003050
	Protein domain specific binding	4	0.0012516
GO Cellular Components	Membrane raft	6	5.15E-08
	Membrane microdomain	6	5.24E-08
	Membrane region	6	6.53E-08
	Extracellular matrix	5	2.40E-05
	Cytoplasmic side of membrane	3	0.000232
	Perinuclear region of cytoplasm	4	0.0012385
	Early endosome	3	0.0017933
	Side of membrane	3	0.00704918
	Postsynapse	3	0.0085988
KEGG Pathway	Focal adhesion	6	3.33E-09
	GnRH signaling pathway	5	4.29E-09
	IL-17 signaling pathway	5	4.53E-09
	VEGF signaling pathway	4	7.46E-08
	Adherens junction	4	1.68E-07
	ErbB signaling pathway	4	3.44E-07
	Rap1 signaling pathway	4	1.21E-05
	MAPK signaling pathway	4	2.59E-05
	TNF signaling pathway	3	5.65E-05
	Platelet activation	3	8.32E-05

### Construction of Component–Target Network

**Figure 5:**
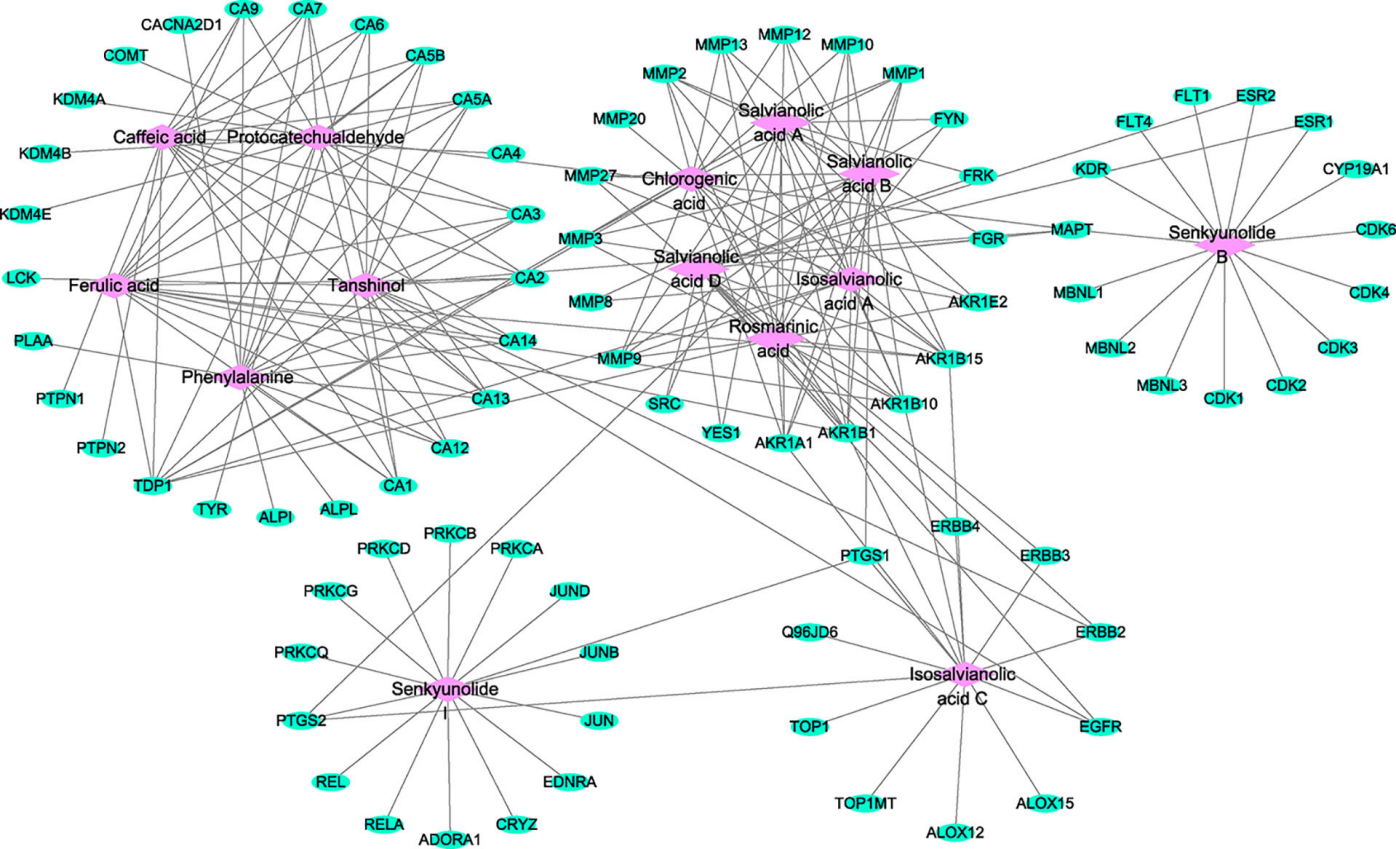
Component–target network for the identified components of GXNT on thrombus. The component–target network was constructed by linking the 14 identified components and their potential targets. The green nodes represent the potential targets and the pink nodes represent the identified components. Edges represent the interactions between the compounds and the targets in the network.

The active component–target network for GXNT on thrombus was constructed using Cytoscape 3.6.1 software. The results showed that a total of 98 nodes and 210 edges were in the identified GXNT component–target network. Network topology analysis showed that the degree and betweenness centrality of Isosalvianolic acid C were the highest (degree=15, betweenness centrality=0.24921196), whereas the degree and betweenness centrality of PTGS1, a target of Isosalvianolic acid C, also ranked among the top (degree=3, betweenness centrality =0.1234151), shown in **Figure 5**.

### Construction of Component–Target–Pathway Network

**Figure 6:**
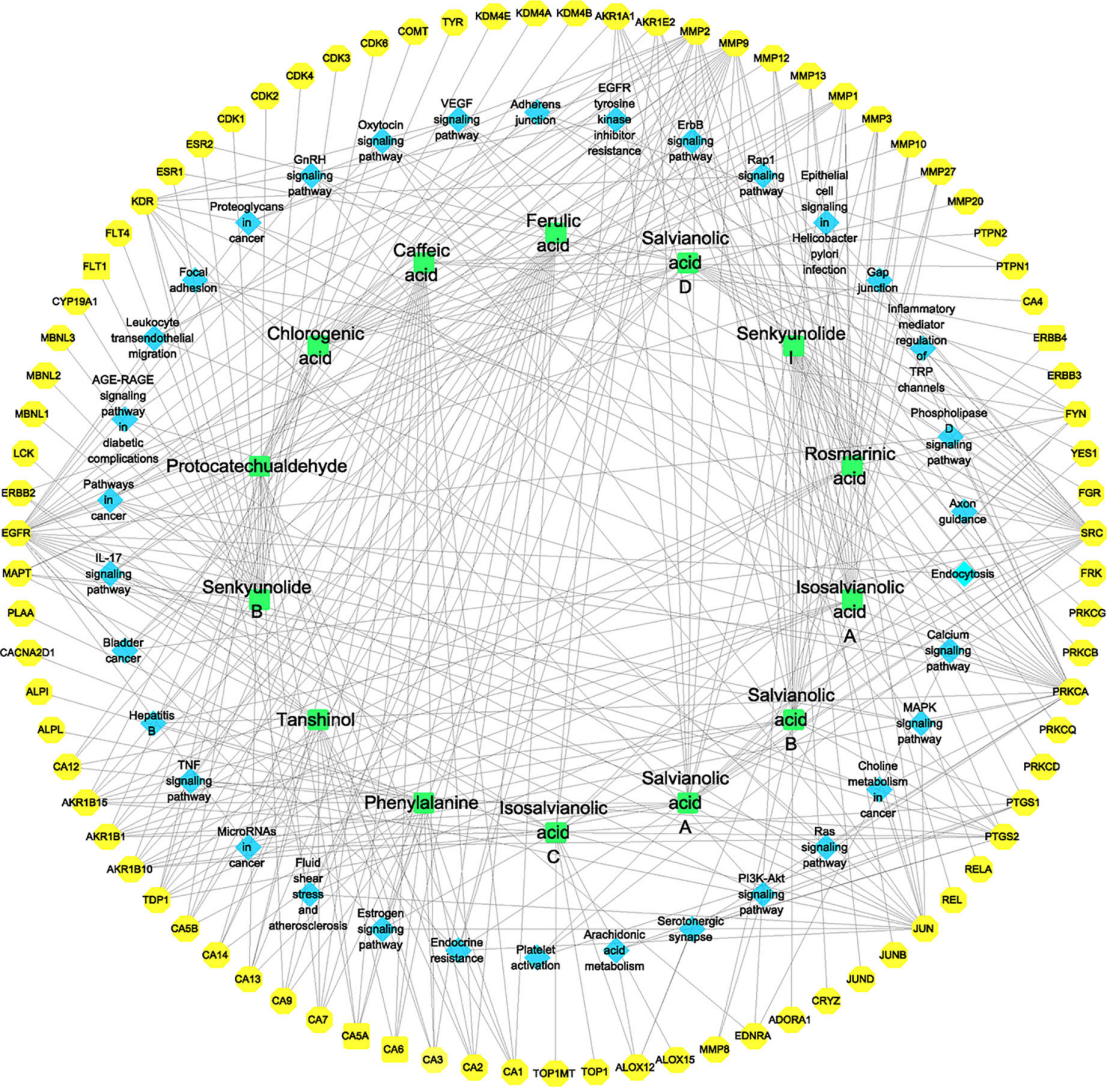
Component–target–pathway network for the identified components of GXNT on thrombus. The green nodes represent the identified components, the blue nodes represent pathways, and the yellow nodes represent targets. Edges represent the interactions between the compounds and the targets in the network.

The Cytoscape software was used to construct the identified component–target–pathway network for GXNT on thrombus. As shown in **Figure 6**, the results showed that multiple targets were associated with multiple components, indicating that different components in GXNT had a synergistic effect in the process of exerting efficacy. The action targets of active ingredients in GXNT were distributed in different pathways and were coordinated with each other, suggesting that the action mechanism of GXNT may be related to the currently known effects of GXNT, such as anti-oxidative stress, anti-inflammation, vascular expansion, anti-platelet aggregation, and protection of vascular endothelium. The component–target–pathway network of GXNT revealed through multiple pathways that GXNT has the characteristic of multiple dimensions and functions for treating thrombotic cardiovascular disease.

### Effects of GXNT on the Length and Weight of Thrombus

**Figure 7:**
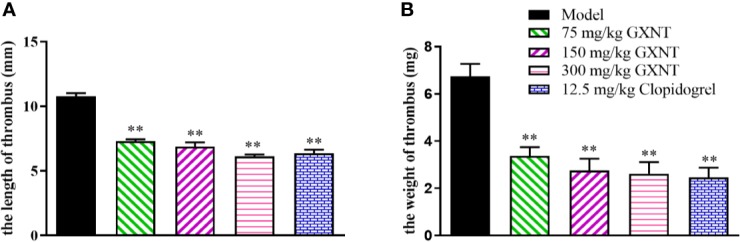
Effects of GXNT on the length and weight of thrombus. **(A)** The length and **(B)** the weight of thrombus were observed after GXNT administration (75, 150, and 300 mg/kg) for 1 h. Data were expressed as the mean ± SEM (± SEM, n=8). **P < 0.01 vs. model group.

We observed changes of thrombus lengths and weights in each group of rats. As shown in **Figures 7A** and **B**, we observed obvious thrombus and significant weight increases in rats of the model group after induction by FeCl_3_. When compared to the model group, GXNT with incremental dosages (75, 150, and 300 mg/kg) could significantly reduce the length and weight of thrombus (*P* < 0.01). In particular, 300 mg/kg of GXNT was superior to clopidogrel in suppressing thrombus length. In addition, the weight of thrombus decreased significantly as the dosage of GXNT increased (*P* < 0.01).

### Effects of GXNT on the Expressions of Fbg, PAI-1, 6-keto-PGF1α, and TXB_2_

**Figure 8:**
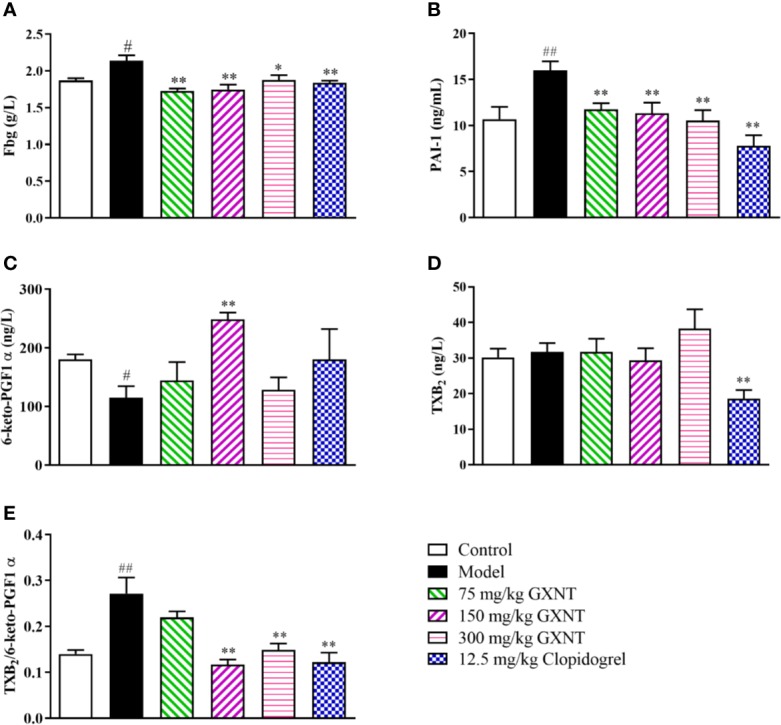
Effects of GXNT on Fbg, PAI-1, 6-keto-PGF1α, TXB2, and TXB2/6-keto-PGF1α. **(A)** Fbg was measured using automatic blood coagulation analyzer. Expression levels of **(B)** PAI-1, **(C)** 6-keto-PGF1α, and **(D)** TXB2 were detected by the enzyme linked immunosorbent assay (ELISA) method. **(E)** The ratio of TXB2/6-keto-PGF1α was then calculated. Data were expressed as the mean ± SEM (± SEM, n=8). ^#^*P* < 0.05, ^##^*P* < 0.01 vs. normal group; *P < 0.05, **P < 0.01 vs. model group.

Fbg is one of the indicators for anticoagulant system activity, and PAI-1, 6-keto-PGF1α, and TXB_2_ are the active markers in fibrinolytic system (**Figures 8A–E**). In **Figures 8A, B**, we can see that the Fbg and PAI-1 expression levels in the model group were significantly increased (*P* < 0.05; *P* < 0.01), compared with those in the control group. The expression levels of Fbg and PAI-1 were reduced in all GXNT groups and the positive group (12.5 mg/kg clopidogrel), compared with those in the model group (*P* < 0.01, *P* < 0.05; *P* < 0.01). As shown in **Figure 8C**, compared to the control group, the expression level of 6-keto-PGF1α in the model group was significantly decreased (*P* < 0.05), and there was no significant change in TXB_2_ expression (*P* > 0.05). Furthermore, the 150-mg/kg GXNT group markedly increased the level of 6-keto-PGF1α (*P* < 0.01) compared to that in the model group, and no significant change was observed in TXB_2_ expression (*P* > 0.05). Meanwhile, the 150- and 300-mg/kg GXNT group significantly decreased the ratio of TXB_2_/6-keto-PGF1α compared with that in the model group (*P* < 0.01, *P* < 0.01; **Figure 8E**).

### Effects of GXNT on Platelet Aggregation

The effect of GXNT on maximum platelet aggregation rate in rats is shown in **Table 6**. The results showed that GXNT could inhibit maximum platelet aggregation rate induced by ADP. Compared with the control group, maximum platelet aggregation rate significantly increased in the model group (*P* < 0.01). Compared with the model group, GXNT with different dosages could decrease maximum platelet aggregation rate to varying degrees. Among them, 150 and 300 mg/kg of GXNT could markedly reduce maximum platelet aggregation rate in rats (*P* < 0.01).

**Table 6:** Effect of GXNT on maximum platelet aggregation rat.

Groups	Drug and Doses	Maximum platelet aggregation rate (%)
Control	10 mL/kg NS	49.33 ± 1.69
Model	10 mL/kg NS	69.17 ± 1.60^##^
The low-dose group	75 mg/kg GXNT	54.83 ± 6.64
The middle-dose group	150 mg/kg GXNT	53.17 ± 2.39**
The high-dose group	300 mg/kg GXNT	52.00 ± 3.34**
The positive group	12.5 mg/kg Clopidogrel	41.50 ± 3.38**

Data were expressed as the mean ± SEM (± SEM, n=8). ^##^P < 0.01 vs. normal group; **P < 0.01 vs. model group.

### Effects of GXNT on Proteins Related to MAPK Signaling Pathway in Platelet

**Figure 9:**
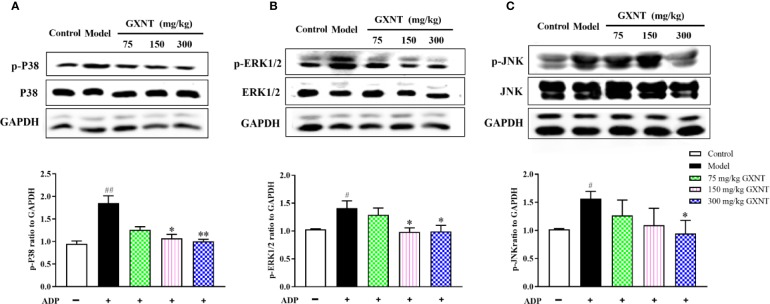
Effects of GXNT on the expressions of p-P38, p-ERK1/2, and p-JNK proteins in platelets. The expressions of **(A)** p-P38, **(B)** p-ERK1/2, and **(C)** p-JNK proteins in platelets were determined by western blotting analysis after GXNT administration (75, 150, and 300 mg/kg). Data were expressed as the mean ± SEM (± SEM, n=3). ^#^*P* < 0.05, ^##^*P* < 0.01 vs. normal group; *P < 0.05, **P < 0.01 vs. model group.

We observed expression changes of p-P38, p-ERK1/2 and p-JNK proteins in the MAPK signaling pathways in each group. From **Figures 9A–C**, it could be seen that after platelet was induced by ADP, phosphorylation levels of P38, ERK1/2, and JNK proteins were significantly increased compared with those in the control group (*P* < 0.01; *P* < 0.05). Compared with the model group, 150 mg/kg GXNT significantly decreased the phosphorylation levels of P38 and ERK1/2 (*P* < 0.05), while 300 mg/kg GXNT significantly lowered the phosphorylation levels of P38, ERK1/2, and JNK (*P* < 0.01; *P* < 0.05).

## Discussion

GXNT consists of extracts from two Chinese herbal medicines, Danshen and Chuanxiong, with the effect of promoting blood circulation and removing blood stasis. The two herbs are compatible with each other to make the blood and qi of human body run smoothly (Zhang, 2017). GXNT has excellent treatment effects on stable or unstable coronary heart diseases and angina pectoris of qi stagnation and blood stasis type triggered by thrombus in the clinic (Huo et al., 2016; Yang et al., 2017; Li et al., 2018). In this study, the pharmacological active substances of GXNT were analyzed, and the action mechanism of these active ingredients was predicted by the approach of network pharmacology, revealing that multiple pathways (such as MAPKs, VEGF, and TNF) are related to the antithrombotic mechanism. The experiments not only further confirmed the antithrombotic effect of GXNT, but also proved that the MAPKs pathway is an important action target. Thus, it showed that network pharmacology could provide high-value insights and reference information for studying the action mechanism of traditional Chinese medicines with complex components.

Thrombosis is an important pathophysiological process involved in various cardiovascular diseases (Otsuka et al., 2016; Ten et al., 2017), and its formation is a complicated process of multifactor participation and gradual development. Abnormal coagulation of blood occurs in the state of flow, due to activation of platelets and clotting factors (Xu et al., 2009). The conditions of thrombosis include vascular intimal injury, changes in blood state, and increased coagulation. Therefore, inhibiting platelet function and preventing blood coagulation can prevent thrombosis. Currently, antiplatelet and antithrombin drugs are often used clinically for treating thrombus, such as platelet cyclooxygenase inhibitor aspirin, platelet ring nucleotide inhibitor dipyridamole, ADP P2Y12 receptor antagonist clopidogrel, and GPIIb/IIIa receptor inhibitor abicximab. However, most of these drugs only have a single target, therefore often require a combination therapy with the risk of causing gastrointestinal hemorrhage (Rocca and Husted, 2016; Dregan et al., 2018). Hence, developing antithrombotic agents with multiple targets and a low risk of hemorrhage from medicinal herbs is of great significance. Our previous studies have shown that GXNT has anti-platelet aggregation and antithrombotic effects (Chen et al., 2005; Wang et al., 2016), which was again validated using the FeCl_3_-induced rat thrombus model in this experiment. Common carotid artery thrombus model in rats induced by FeCl_3_ is widely used in the preparation of arterial models and in the research of antithrombotic drugs (Kurz et al., 1990). The results of this experiment showed that the length and weight of thrombus were increased significantly after the formation of common carotid artery thrombus in rats. The administration of GXNT could significantly reduce the length and weight of thrombus. Furthermore, the antithrombotic effect was dose-dependent, indicating that GXNT is strongly resistant to FeCl_3_-induced thrombus. Fe^3+^ can damage vascular endothelial cells and cause platelet activation and aggregation, due to a joint action of the coagulation system and the hemolysis system. When endothelial cells are damaged, the internal and external coagulation systems are activated. Thrombin activates platelets and converts Fbg to fibrin, which in turn activates the coagulation response system. Thus, Fbg plays a major role in the blood coagulation process, while PAI plays a major role in regulating plasma fibrinolytic activities. In addition, TXA_2_ is a biologically active substance that strongly promotes vasoconstriction and platelet aggregation, whereas PGI_2_ can dilate blood vessels and increase platelet cAMP to inhibit platelet aggregation. The dynamic balance between TXA_2_ and PGI_2_, i.e. ratio of TXA_2_/PGI_2_, plays an important role in maintaining the function of platelet and vessels and the regulation of thrombosis. Because of the instability of TXA_2_ and PGI_2_, their stable metabolites (TXB_2_ and 6-keto-PGF1α) were used as detection indicators in this study (Xie et al., 2017; Cui et al., 2018). Our results showed that the level of Fbg was increased significantly in the thrombus model rats, whereas GXNT reduced the levels of Fbg and PAI, and regulated the balance of 6-keto-PGF1α and TXB_2_. The results demonstrated that GXNT could ameliorate the hypercoagulable state of the body blood, and maintain the balance of the body's coagulation system and anticoagulant system, thereby achieving an antithrombotic effect.

In this study, 14 potential active components were identified using LC-MS technology. In specific, 8 were from Danshen, 4 were from Chuanxiong, and 2 were common components in both. In general, most compounds had a certain response in both positive and negative modes, but the negative mode response was slightly higher than the positive mode response. This is mainly due to the fact that GXNT contains more salvianolic acids from Danshen, which is more prone to give protons in the negative mode to obtain higher ion response. Nevertheless, some of the compounds only responded in the positive mode, mainly due to the fact that the lactones from Chuanxiong could only bind to protons and are difficult to give protons. Among these components, salvianolic acid B, salvianolic acid A, ferulic acid, chlorogenic acid, caffeic acid, rosemary acid, tanshinol, and protocatechualdehyde have already shown antithrombotic effects to some extent in previous studies (Jiang et al., 2005; Moon et al., 2012; Chen et al., 2018b). The modern pharmacological research showed that tanshinol could dilate blood vessels, promote fibrinolysis, reduce blood viscosity, and promote local blood circulation, thereby exerting antithrombotic effects (Dang et al., 2015). Salvianolic acid could inhibit arterial thrombosis by restraining platelet adhesion, aggregation, and downstream Ca^2+^ and cAMP signaling pathways (Hong et al., 2016). In addition, the antithrombotic effect of chlorogenic acid may be closely related to the adenosine A_2A_ receptor/adenylate cyclase/cAMP/PKA signaling pathway (Fuentes et al., 2014). Thus, it can be seen that the multi-level and multi-target effects were determined by multiple active ingredients of GXNT. We will confirm whether these active ingredients have synergistic effects in our future experimental studies.

To understand the antithrombotic mechanism of GXNT, we used network pharmacology for the analysis. It turned out that the 14 components in GXNT were closely related to 83 targets, of which 17 potential antithrombotic targets were obtained through functional enrichment analysis and are involved in pathways including MAPK signaling pathway, TNF signaling pathway, and VEGF signal transduction pathway. Based on this finding, MAPKs signaling pathway, which is known to be strongly linked to thrombotic diseases (Endale et al., 2012; Lien et al., 2017; Manne et al., 2018), was selected to verify the antithrombotic mechanism of GXNT. The MAPKs signaling pathway exists in most cells. Recent studies have shown that the MAPKs signaling pathway is an important platelet activation pathway that can be activated by collagen and thrombin to mediate platelet deformation, adhesion, and aggregation reaction, thereby participating in thrombus formation (Lien et al., 2017; Manne et al., 2018). The signaling pathway includes three subfamilies: extracellular signal regulated protein kinase (ERK), c-Juc amino-terminal kinase (JNK), and p38 protein kinase (p38 MAPK) (Lanna et al., 2017). The study by Yacoub (Yacoub et al., 2006) has shown that the activation of ERK and p38 MAPK plays an important role in the release of TXA_2_ mediated by PKC. After being stimulated from collagen and thrombin, the phosphorylation level of PKCδ was increased in platelets, leading to an activation of p38 MAPK and ERK as well as a release of TXA_2_. Simultaneously, the p38 MAPK signaling pathway is involved in the synthesis of platelet cells and backbone proteins. Activated p38 MAPK can induce regeneration and reorganization of actin and dynamic changes of platelet cytoskeleton *via* regulating the activity of heat shock protein (HSP27) and the level of downstream vasodilation-stimulated phosphoprotein (VASP), and can therefore cause platelet degeneration and promoting thrombosis (Mazharian et al., 2007). It has been found that JNK1 is involved in platelet aggregation and thrombosis. An *in vitro* study using rats with JNK1 deficiency (Adam et al., 2010) has shown an activation of integrin αIIbβ3 by PKC, a reduction of platelet aggregation, and an occurrence of platelet secretion disorder, indicating that JNK1 may play a key role in platelet biology and thrombosis. Moreover, some active components in GXNT such as caffeic acid (Lu et al., 2015), salvianolic acid B (Li et al., 2010), and ferulic acid (Hong et al., 2016) may be involved in the regulation of MAPKs signaling pathway. In our study, platelet aggregation was promoted by ADP as the inducer, and the phosphorylation levels of ERK, p38, and JNK proteins in MAPKs signal pathway of platelets were determined by Western Blot. The results showed that GXNT inhibited ADP-induced platelet aggregation. In addition, the phosphorylation levels of p38 MAPK, ERK, and JNK in rat platelets were all significantly increased compared to those in the control group, suggesting that ADP may affect platelet function by influencing the phosphorylation levels of p38 MAPK, ERK, and JNK proteins in the MAPKs signaling pathway. After the intervention with GXNT, the phosphorylation levels of p38 MAPK, ERK, and JNK proteins were all decreased, especially the phosphorylation level of p38 MAPK protein. Hence, GXNT had clear anti-platelet aggregation and antithrombotic effects, which may be achieved through reducing the phosphorylation levels of p38 MAPK, ERK, and JNK in MAPKs signaling pathway.

## Conclusion

In conclusion, 14 active ingredients of GXNT were identified in this study, and the antithrombotic and antiplatelet aggregation effects of GXNT were further confirmed. Through the approach of network pharmacology, 34 signal pathways were predicted to be involved in thrombus (including MAPKs, VEGF, and TNF), and the role of MAPKs signal pathway in thrombotic diseases was verified. We further showed that the antithrombotic mechanism of GXNT may be associated with suppressing the phosphorylation of p38MAPK, ERK, and JNK in the MAPKs signaling pathway. The results from this study provided a reference for future studies on the action mechanism of GXNT for treating thrombotic diseases, as well as demonstrated that network pharmacology approaches can be used to predict the action mechanism of traditional Chinese medicine with complex components.

## Data Availability Statement

All datasets generated for this study are included in the article/supplementary material.

## Ethics Statement

The animal study was reviewed and approved by Institutional Animal Care and Use Committee of Zhejiang Chinese Medical University.

## Author Contributions

M-LC contributed to the design concepts of this whole study. X-HY, Z-WZ, M-LW, and Y-YL carried out the study and collected important background information. M-LW, Y-YL, Q-QY, and Y-SW drafted the manuscript. Q-QY and Q-XM carried out literature search, data acquisition and analysis, and manuscript revision and edition. Q-YS helped perform the analysis with constructive discussions. All authors have read and approved the content of the manuscript.

## Funding

This research was funded by Key Projects of Zhejiang Provincial Administration of Traditional Chinese medicine (2015ZZ009) and Zhejiang Science and Technology Department Public Welfare (Experimental Animal Platform) Project (2018C37129).

## Conflict of Interest

M-LW, X-HY, Z-WZ, were employed by company Chiatai Qingchunbao Pharmaceutical Co., Ltd.

The remaining authors declare that the research was conducted in the absence of any commercial or financial relationships that could be construed as a potential conflict of interest.
